# Autopsy registry can facilitate COVID‐19 research

**DOI:** 10.15252/emmm.202012885

**Published:** 2020-07-03

**Authors:** Saskia von Stillfried, Roman David Bülow, Rainer Röhrig, Ruth Knüchel‐Clarke, Peter Boor, the DeRegCOVID, Pauline Tholen, Barbara Nöthel, Jan Wienströer, Raphael Majeed, Stefan Uhlig, Danny Jonigk, Hans‐Ulrich Holtherm, Karl‐Friedrich Bürrig, Gustavo Barreton

**Affiliations:** ^1^ Institute of Pathology RWTH Aachen University Hospital Aachen Germany; ^2^ Institute of Medical Informatics RWTH Aachen University Hospital Aachen Germany

**Keywords:** Microbiology, Virology & Host Pathogen Interaction

## Abstract

The WHO declared the global outbreak of SARS‐CoV‐2 a pandemic on March 11, 2020, and “call(ed) on all countries to exchange country experiences and practices in a transparent and timely way” (http://www.euro.who.int/en/health-topics/health-emergencies/pages/news/news/2020/03/who-announces-covid-19-outbreak-a-pandemic). To date, many medical societies have announced their intention to collect and analyze data from COVID‐19 patients and some large‐scale prospective data collections are already running, such as the LEOSS registry (Lean European Open Survey on SARS‐CoV‐2 Infected Patients) or the CAPACITYCOVID registry (registry of patients with COVID‐19 including cardiovascular risk and complications). The necessity to mobilize and harmonize basic and applied research worldwide is of utmost importance (Sansonetti, 2020).

## Graphical abstract

This commentary describes the first nation‐wide registry of COVID‐19 autopsies. Autopsies are an essential tool for the understanding of infectious diseases, and the initiative presented here will help harmonizing and accelerating research on COVID‐19.

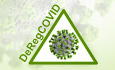

The autopsy is an important instrument to understand the pathogenesis of diseases, including infectious diseases and novel pathogens like Ebola, SARS, or SARS‐CoV‐2 (Nicholls *et al*, 2003; Mari Saez *et al*, 2015). The first autopsy studies already suggested important disease mechanisms in fatal COVID‐19 cases with potential therapeutic implications. These include increased thromboembolism and vascular dysfunction (Lax *et al*, 2020; Menter *et al*, 2020; Wichmann *et al*, 2020), infection of endothelial cells (Varga *et al*, 2020), viral spread in different organs (Puelles *et al*, 2020), or the pathological mechanisms of lung injury (Ackermann *et al*, 2020; Schaller *et al*, 2020). Despite this, no ongoing registry gathered autopsy data, and no specific autopsy registry existed until our recent initiative.

We have established and launched the worldwide first national registry of COVID‐19 autopsies in Germany, the DeRegCOVID (Fig 1). The registry was launched in cooperation with the German professional societies of pathology and supported by the Federal Ministry of Health. It aims at gathering data on potentially all autopsies in Germany in a factually anonymized manner. The main focus is on the pathological findings and cause of death at autopsy according to the WHO recommendations, flanked by clinical disease course of the infection, known preexisting conditions, and basic demographic data. Central data curation ensures high data quality. The registry will serve as a central hub for data analyses, the results of which will be reported to the professional societies and the Federal Ministry of Health, and jointly communicated to the public. Detailed data on available biosamples, which remain decentralized with each participating center, are also gathered. This allows the registry to serve as an honest broker for national and international research inquiries, facilitating and supporting research, as recently shown in the analyses of the pulmonary involvement in COVID‐19 (Ackermann *et al*, 2020). The available samples from virtually all organs will enable the analyses of organ‐specific effects of COVID‐19. Interoperability with other registries, e.g. LEOSS, is being implemented. The registry also provides detailed standard operating procedures (SOP) and supports nationally and internationally centers and societies in performing COVID‐19 autopsies. Despite a non‐mandatory participation in the registry, the contribution of centers across Germany, both academic and non‐academic, is already high. Each center gets its profile and login data for the online secure platform for sustainable and prospective data reporting and management. The platform can be modularly expanded, include additional data, serve as a model for other national registries, or gather data on an international scale.

**Figure 1:**
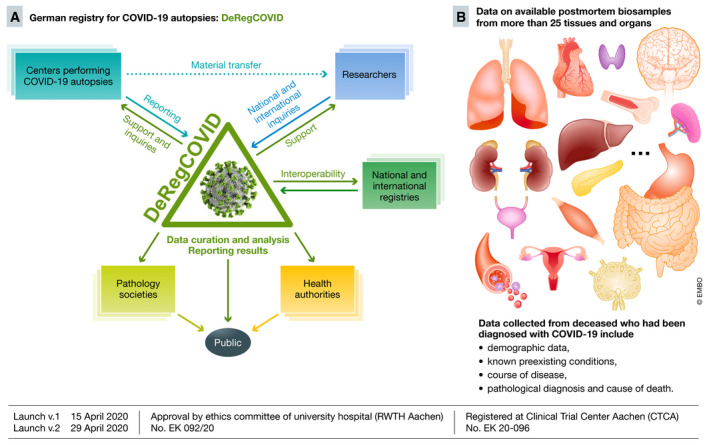
The German Registry for COVID‐19 Autopsies (DeRegCovid) (A) DeRegCOVID workflow: The main aim of the registry is to centrally gather possibly all autopsy data from Germany. The registry supports centers in all questions related to COVID‐19 autopsies, e.g. providing detailed standard operating procedures. The biomaterial remains decentralized with each center. The centers report data to the registry. The registry reports the data to the pathology societies, the health authorities, i.e. German Federal Ministry of Health and Robert Koch Institute, and, jointly with these institutions, to the public. The registry also serves as an honest broker mediating national and international research inquiries to centers with available material. (B) Available material and data: More than 25 different tissues are available, mainly formalin‐fixed paraffin‐embedded (FFPE), but several centers also have unfixed frozen samples. Gathered data mainly focus on the pathological diagnosis derived from the autopsy. The registry was first launched on April 15, 2020. Green lines represent the tasks performed by the registry.

We hope that this registry will serve as an example of how to further strengthen structured research on COVID‐19, potentially sparking and accelerating similar initiatives on an international level.

## Conflict of interest

The authors declare that they have no conflict of interest.
